# Cross‐Sectional and Longitudinal Associations of the Pupillary Light Response with Mild Cognitive Impairment

**DOI:** 10.1002/alz70856_103754

**Published:** 2025-12-24

**Authors:** Matthew S. Panizzon, Eric L. Granholm, Kristine C Dell, Francesca Lopez, Chandra A. Reynolds, Carol E. Franz, William S. Kremen, Jeremy A. Elman, The Vietnam Era Twin Study of Aging

**Affiliations:** ^1^ Center for Behavior Genetics of Aging, University of California, San Diego, La Jolla, CA, USA; ^2^ Ceneter for Behavior Gentics of Aging, La Jolla, CA, USA; ^3^ University of California San Diego, La Jolla, CA, USA; ^4^ University of California, San Diego, San Diego, CA, USA; ^5^ Unversity of California San Diego, La Jolla, CA, USA; ^6^ University of Colorado Boulder, Boulder, CO, USA; ^7^ Institute for Behavioral Genetics, University of Colorado Boulder, Boulder, CO, USA

## Abstract

**Background:**

Pupillary light response (PLR) alterations have been observed in individuals with Alzheimer's disease and related dementias (ADRD). Little is known about the predictive utility of the PLR as a biomarker for ADRD or general cognitive decline. In the present study we determined if the PLR was cross‐sectionally associated with mild cognitive impairment (MCI) during late midlife, as well as whether it was predictive of conversion to MCI over a 6‐year follow‐up.

**Method:**

Data were utilized from waves 2 (mean age=61.7) and 3 (mean age=67.6) of the Vietnam Era Twin Study of Aging (VETSA), a population‐based cohort of men. MCI status was based on Jack‐Bondi criteria with impairment defined as performance >1.5 SD below the mean on 2+ tests within a cognitive domain. Pupil response was assessed at wave 2 with NeurOptics PLR‐200 pupillometers. PLR measures included peak constriction amplitude, constriction latency, mean constriction velocity, recovery time, and dilation velocity. All analyses controlled for age, race/ethnicity, initial pupil diameter, device, total number of anticholinergic medications, and clustering of twin pairs. Participants reporting a significant history of eye injury, eye surgery, ocular medications, or glaucoma were excluded. Longitudinal analyses were restricted to participants designated as cognitively normal at the first assessment.

**Result:**

In cross‐sectional analyses (*N* = 1070) reduced peak constriction amplitude was significantly associated with higher likelihood of having MCI (OR = 1.76; 95% CI = 1.22; 2.53). The association was observed for both amnestic and non‐amnestic MCI subtypes. In longitudinal analyses (*N* = 734) dilation velocity was significantly associated with conversion to amnestic MCI at the 6‐year follow‐up (OR = 1.97; 95% CI = 1.22; 3.17).

**Conclusion:**

These results demonstrate that aspects of the pupillary light response are significantly associated with MCI at late midlife and predictive of conversion to amnestic MCI over a 6‐year follow‐up, suggesting its utility as an easily acquired, non‐invasive biomarker for risk of cognitive decline and ADRD prior to overt impairment. The observed associations are consistent with either hypoactivity in parasympathetic anticholinergic systems and/or hyperactivity in locus coeruleus–norepinephrine systems in preclinical AD, and possibly dysfunction in photosensitive melanopsin expressing retinal ganglion cells in preclinical AD.

